# Screening of volatile organic compounds (VOCs) from liquid fungal cultures using ambient mass spectrometry

**DOI:** 10.1007/s00216-023-04769-6

**Published:** 2023-06-30

**Authors:** Daniel Heffernan, Melania Pilz, Marco Klein, Martina Haack, Alan M. Race, Thomas Brück, Farah Qoura, Nicole Strittmatter

**Affiliations:** 1 Department of Biosciences, TUM School of Natural Sciences, Technical University of Munich (TUM), Garching, Germany; 2 Department of Chemistry, TUM School of Natural Sciences, Technical University of Munich (TUM), Garching, Germany; 3 Institute of Medical Bioinformatics and Biostatistics, University of Marburg, Marburg, Germany

**Keywords:** Volatile organic compounds (VOCs), Fungi, Dielectric barrier discharge ionisation (DBDI), Ambient mass spectrometry, Headspace analysis

## Abstract

The potential of fungi for use as biotechnological factories in the production of a range of valuable metabolites, such as enzymes, terpenes, and volatile aroma compounds, is high. Unlike other microorganisms, fungi mostly secrete secondary metabolites into the culture medium, allowing for easy extraction and analysis. To date, the most commonly used technique in the analysis of volatile organic compounds (VOCs) is gas chromatography, which is time and labour consuming. We propose an alternative ambient screening method that provides rapid chemical information for characterising the VOCs of filamentous fungi in liquid culture using a commercially available ambient dielectric barrier discharge ionisation (DBDI) source connected to a quadrupole-Orbitrap mass spectrometer. The effects of method parameters on measured peak intensities of a series of 8 selected aroma standards were optimised with the best conditions being selected for sample analysis. The developed method was then deployed to the screening of VOCs from samples of 13 fungal strains in three different types of complex growth media showing clear differences in VOC profiles across the different media, enabling determination of best culturing conditions for each compound-strain combination. Our findings underline the applicability of ambient DBDI for the direct detection and comparison of aroma compounds produced by filamentous fungi in liquid culture.

**Graphical abstract:**

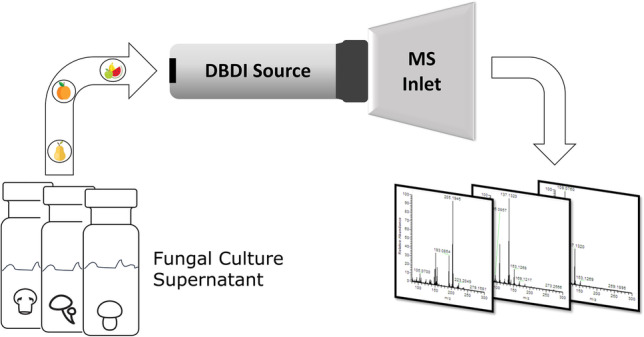

**Supplementary Information:**

The online version contains supplementary material available at 10.1007/s00216-023-04769-6.

## Introduction

Fungi pose a great source for the natural production of high-value metabolites, such as enzymes, terpenes, and volatile aroma compounds (VOCs) [1]. Volatile compounds are often utilised in intercellular communication (quorum-sensing) [2–4] between fungal cells, or fungal interactions between fungi and different species, such as insects [5–7], plants [8–12], and other microbes [13–15]. Differently from bacterial producers, fungi mostly secrete secondary metabolites into their culture medium [16]. This facilitates the extraction of the compounds, as the cells do not have to be disrupted, decreasing the amount of unwanted intracellular contaminants within the product. Among biotechnologically produced high-value compounds, aroma substances are used in a variety of industrial sectors, such as food [17, 18] and animal feed [19, 20], as well as personal care [21] and medicinal products [22]. Within the food and beverage industry, companies rely on flavourings in drinks, dairy, bakery, and confectionary products. With a market value of $12,712.7 million in 2020, the importance of natural and artificial food flavourings has risen in the past decades on an international scale, with the Asia-pacific region at the forefront of the expansion [23]. During the last decades, consumer awareness for biologically sourced products [24] increased the need for alternative production methods of natural compounds without the use of unethical production practices or genetic engineering (non-GMO) [25]. Furthermore, chemically synthesised compounds (mostly of petrochemical origin) face a stigma from many consumers who are averse to artificial flavouring and prefer purchasing naturally flavoured products [17, 24, 26]. Sustainable production of natural flavour compounds can be designed based on circular bioprocesses utilising industrial and agricultural waste streams [27, 28]. By-products resulting from the production, such as mycelial residue, can be used as protein-rich feed additives in ecological agriculture [29].

In their natural environment as e.g. plant pathogens, the main VOCs of fungal aroma producers include a variety of aldehydes, ketones, alcohols, lactones, and terpenoids [30–32]. The intensity of secretion and variety of VOCs is dependent on the substrate they infect, such as type of fruits. The natural VOCs of their host plants were found to be altered and/or intensified by fungal pre- and even more post-harvest infections [33]. The secretion of fungal VOCs has been proven beneficial for agricultural applications, e.g. for the stimulation of plant growth [10–12] or as a biocontrol agent [10, 11, 34, 35]. Following the characterisation of secretion and production pathways, media and cultivation optimisation are necessary for high yield production of aroma compounds. The tailored production of natural aroma compounds is dependent on various cultivation parameters, such as the supplied nutrients and carbon and nitrogen sources, as well as incubation time, pH, and temperature conditions (Table S1). The impact of various carbon and nitrogen sources on the aroma profile of *Ceratocystis moniliformis* was originally reported by Lanza et al. (1979) [36]. A spectrum of tropical and citrusy fruit flavours was achieved with the right supplementation enabling the fine tuning of flavour using one production organism. Supplementation with precursors including intermediary metabolites within biosynthetic pathways enables efficient VOC secretion as seen for e.g. α-terpineol production. Using similar strategy, Rottava et al. (2010) described the conversion of (−)-β-pinene and R-(+)-limonene into α-terpineol among other minor VOCs, such as trans-pinocarveol, pinocamphone, and fenchol by *Aspergillus* sp. and optimisation of the biotransformation process (2011) [37, 38]. Apart from the production of specific flavour compounds or tailored bouquets, fungi, such as *Aspergillus niger* PW-2, are applied during the fermentation of green tea leaves, altering the flavour of the product [39]. Other biotechnologically relevant fungal producers are e.g. ascomycetes from the genus *Neurospora* [40, 41]*.* They produce VOCs, such as 1-octen-3-ol [42], which is widely utilised as a mushroom aroma, among other industrially relevant compounds.

Soft Ionisation by Chemical Reaction Transfer (SICRIT; Plasmion, Augsburg, Germany) is a commercial plasma-based soft ionisation source based on dielectric barrier discharge ionisation (DBDI). DBDI is an ambient ionisation source introduced in 2007 by Na et al. [43] and demonstrates ionisation characteristics similar to atmospheric pressure chemical ionisation (APCI). The term dielectric barrier discharge refers to a type of gas discharge where a low-temperature plasma is produced by discharge between two electrodes separated by a dielectric barrier via the application of a high-voltage alternating current with frequencies in the kHz or MHz range. Primary reactive species like N_2_^+^, nitrogen oxides, or excited nitrogen are formed depending on the discharge gas, which go on to chemically ionise analytes for subsequent mass spectrometric analysis [44]. The ionisation process in the plasma is complex and often not completely known due to the many different constituents such as positively and negatively charged ions, electrons, atoms, free radicals, and photons [45]. Despite this, most low-temperature plasma ionisation produces molecular ions (e.g., M^−^, MH^+^) with little fragmentation [46, 47] with the exception of saturated alkanes, which produce characteristic oxidised ions with the generic formula [M-(2n-1)H+*m*O]^+^ [44].

The analysis of microbial VOCs has almost exclusively been reported by using gas chromatography-mass spectrometry (GC-MS) [48]. While this method has become the standard for microbial VOC analysis, it is time consuming, requires skilled operators, and is relatively expensive with regard to consumables. DBDI-MS methods have been receiving increasing interest due to offering solutions to these problems. Minimal sample preparation using no expensive reagents and soft ionisation that generates ions that are representative of the original structure of the initial analytes are major hallmarks of the methodology. The analysis of gaseous species via DBDI has been demonstrated for many applications, from the detection of chemical warfare agents [49] through engine exhaust aerosols [50], pharmaceutical products [51], to VOCs [52].

The objective of this study was to assess the suitability of the DBDI method for the screening of volatile compounds produced by filamentous fungi in liquid culture as an alternative approach for culturing condition optimisation. For this, we investigated different setups for sample introduction, spectral appearance of analytes and spectral stability, carry-over, and equilibration settings on a mixture of standards before applying the best experimental settings to the analysis of fungal cultures in liquid medium. Eight aroma standard compounds were chosen in order to optimise the analysis conditions. The chosen standard solutions were based on a preliminary literature research on VOCs produced by the genus chosen in this study as seen in Supplementary Table S1. Most commonly found VOCs for *Ceratocystis* sp. are ethanol and ethyl acetate [36, 53], which is supported by the GC-FID/GC-MS results. Ethanol could only be detected by GC-FID, not by GC-MS, as the cut-off for detection starts at 50 *m*/*z* (results not shown in this study). Further substances detected include isoamyl acetate and 2-methyl-1-butanol. As seen for *Neurospora* sp., 1-octen-3-ol and ethyl hexanoate are, apart from ethanol and ethyl acetate, among the most commonly detected VOCs for this genus [40, 42]. These substances could also be detected using GC-FID/GC-MS (results not shown in this study). *Aspergillus niger* strains were found to produce α-terpineol, trans-pinocarveol, pinocamphone, and fenchol in previous publications [37, 38], as well as 1-octen-3-ol, geraniol, and nonanal among others [39]. None of the previously mentioned compounds for *Aspergillus* sp. VOCs could be detected with GC-FID/GC-MS in our current setup.

## Materials and methods

### Chemicals

Isopentyl acetate, isobutyl acetate, 2-phenylacetate, 2-phenylethanol, 2-methyl-1-butanol, ethyl acetate, citral, and beta-citronellol were purchased from Sigma-Aldrich (Merck). Methanol and water were LC-MS grade and purchased from VWR. Two standard stock mixes were produced: Mix 1: isopentyl acetate, isobutyl acetate, 2-phenylacetate, 2-phenylethanol, 2-methyl-1-butanol, ethyl acetate; mix 2: isopentyl acetate, isobutyl acetate, 2-phenylacetate, 2-phenylethanol, 2-methyl-1-butanol, ethyl acetate, citral, and β-citronellol. All pure standards were in liquid aggregate form at room temperature. Five microliters of each pure standard was mixed into 100 mL of 10% methanol solution in water or 100 mL 100% water and dilutions by a factor of 3 produced from there (0.3, 0.09, 0.027, 0.0081); corresponding concentrations in mol/L are given in Table S2. For the lowest dilution factor, concentrations in the 2–4 μM range are achieved. The ingredients, sum formulas, and structures of the standards are given in Table 1.

**Table 1:**
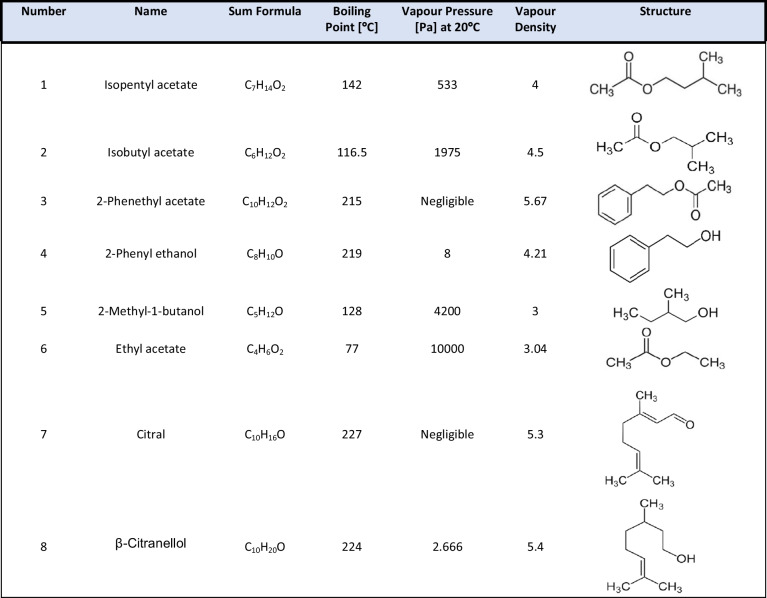
VOC standards used in this study

### Fungal culture

A total of 13 strains known to produce volatile aromatic compounds were chosen for the method development of aroma compound detection (see Table S3 for list). The ascomycete fungi belonging to *Aspergillus* sp., *Ceratocystis* sp., and *Neurospora* sp. were supplied by the German Collection of Microorganisms and Cell Cultures GmbH (DSMZ, Brunswick, Germany) and the Westerdijk Fungal Biodiversity Institute (Utrecht, Netherlands) (CBS strain database) and cultivated in three complex media for aroma production. Potato medium with 4 g/L or 20 g/L potato extract and YPD with 10 g/L yeast extract and 20 g/L peptone were supplemented with 2% glucose. The 30-mL cultures were incubated at 28 °C and 120 rpm for 5 days before harvesting and sterile filtration of the supernatant. The growth conditions for all fungi were the same. The growth morphology for the tested fungal species is different based on the strain within the same medium, and so is their biomass and natural product secretion.

### Sample preparation

All experiments were performed with 2 mL of standard mix or filtered culture supernatant in 20 mL headspace vials with screw cap and septum. Sample vials were sealed upon collection of the supernatant and only re-opened when analysis was being performed. Use of silicone-containing septa was avoided as these may lead to strong polysiloxane contamination peaks in DBDI analysis. Instead, silicone-free TEF rubber septa were purchased from VWR. Unless stated otherwise, the sealed sample vials were incubated at 60 °C in an Ohaus dry block heater (4 blocks) for 15 min. In order to keep the method compatible with the envisioned screening application, we kept this step as simple as possible by only heating the solutions in a water bath without further shaking or stirring the solutions. Once removed from the heating block, vials were analysed immediately.

### DBDI-MS analysis

All spectra were acquired in positive ion mode by analysing pure and diluted standard solutions by placing them directly in front of the DBDI source (SICRIT, Plasmion, Augsburg, Germany) inlet for several seconds. Room temperature during analysis was typically between 19 and 22 °C, with humidity ranging from 30 to 40%. Background atmosphere and sample headspace was aspirated at a rate of 1 m^3^/min as per the Q-Exactive manual. Three different experimental setups were tested with 2 mL of standard solution (compounds **1**–**8** in 10% methanol, 5 μL/100 mL each) in 20-mL headspace vials each for their practicability. Schemes of the setups are shown in Figure S1 with selected total ion chromatograms in Figure S2. Method A (Figure S1A) consists of transfer of the headspace from the sealed vial using a 25-cm-long 1/16″ OD PTFE polymer tubing inserted through the septum, method B (Figure S1B) consists of ambient sampling where the open vials were placed directly in front of the DBDI source, and method C (Figure S1C) in which 2 mL of the headspace is removed via a gastight syringe through the septum of the closed vial and the headspace is subsequently directly infused into the DBDI source using a syringe adapter at a speed of 0.5 mL/min. Each measurement was repeated five times and standard deviations calculated to determine the most stable analysis method. Method B was found superior and was used for further experiments and is described below. Photographs of setup B can be found in Figure S1D-E.

For ambient sampling, the samples were clamped at a 30° angle directly under the inlet capillary (as shown in Figure S1E) while a background blank was recording. Once in position, the tripod was shortly turned to the side to remove the cap, the vial immediately placed back into position, and spectra were recorded for a defined and constant amount of time. The vial was then removed and further background was recorded to allow for any possible carry-over to dissipate. For assessment of spectral features and carry-over, linearity of the data, and best data extraction intervals, we have used the following timings: background spectra 3 min, active sampling for 5 min, background for 5 min. Total run duration was 13 min per sample. For optimisation of equilibration conditions and fungal analysis, we have used the following timings: background spectra 1 min, active sampling for 1 min (data extraction for the period of 30–60 s only), background for 2 min. Total run duration was 5 min per sample. To assess the linearity of the data and best data extraction intervals, a calibration experiment was performed between 0.3 and 0.0081 dilution and in triplicate to assess the linearity of the method for different standard compounds using ambient sampling.

Plasma settings were 1500 V at 15 kHz. The manufacturer recommends a range of 1200–1500 V; however, no signal was observed below 1400 V. Similar settings were used in previous other studies, leading to successful ionisation of molecules up to the size of glycerophospholipids (*m*/*z* 900) [54, 55] and which were recently found optimal source parameters for a set of different lipid species, with different RF frequency settings not showing a noticeable influence on intensity [56]. SICRIT ion source was mounted onto a Q-Exactive (Thermo Fisher Scientific, Bremen, Germany) mass spectrometer operated at a resolution of 70,000 at *m*/*z* 200. Capillary temperature was 200 °C, maximum injection time was 50 ms, and S-Lens RF setting was 50. Spectra were acquired at mass ranges between 60 and 500 Da for full-scan spectral acquisition and 50–220 Da for acquisition of fragmentation spectra. Fragment spectra were recorded at an intermediate HCD setting of 30.

### Data analysis

Data analysis was performed using QualBrowser (Thermo Scientific) and Excel (Microsoft).

## Results

### Spectral appearance

**Fig. 1:**
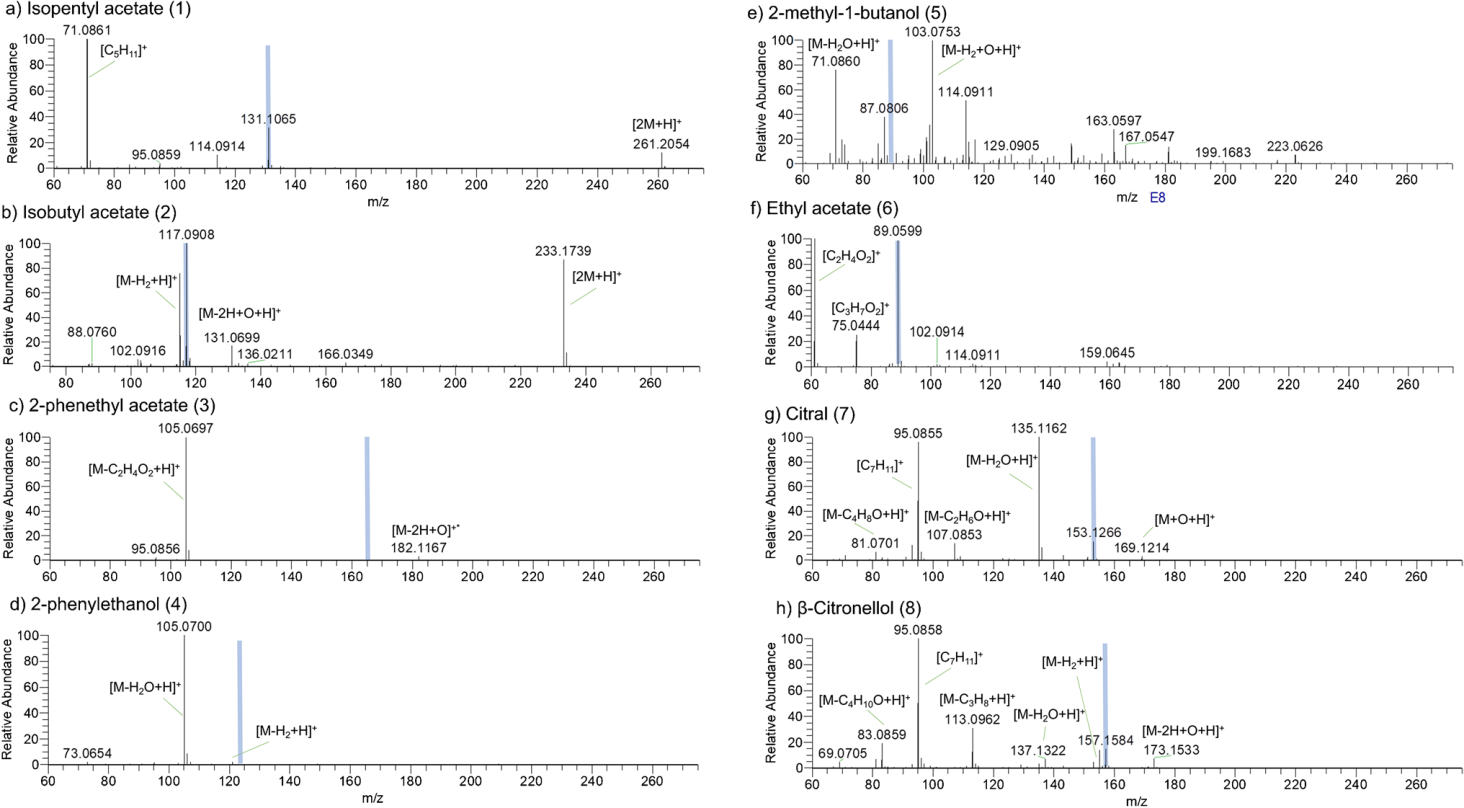
Mass spectra obtained for concentrations of 5 μL/100 mL for compounds **1**–**8** using ambient sampling (method B). (**a**) isopentyl acetate **1**, (**b**) isobutyl acetate **2**, (**c**) 2-phenethyl acetate **3**, (**d**) 2-phenyl ethanol **4**, (**e**) 2-methyl-1-butanol **5**, (**f**) ethyl acetate **6**, (**g**) citral **7**, and (**h**) β-citronellol **8**. Light blue lines indicate theoretical position of the [M+H]^+^ peak. Identified fragments are assigned based on the monoisotopic mass M or by giving their formula

We analysed the ionisation behaviour of different standards that we suspected as possible compounds present among fungal aroma compounds (see Table S1). The ionisation of all analysed compounds was superior in positive ion mode which led us to abandon negative ion mode for further studies. DBDI is expected to be a soft ionisation method producing spectra mainly featuring protonated ions [47, 54–56]. While we have used it with settings in line with previous studies of the same commercial unit, we have detected extensive fragmentation for all of our small organic analytes (see Fig. 1, Table S4 for a list of all peaks observed for each individually analysed standard). For compound **5**, no quasi-molecular ion was observed at all while for **1**, **3**, **4**, **6**, **7**, and **8**, the molecular ion peak was not the base peak with relative intensities being 38%, 0.26%, 0.01%, 95%, 19%, and 20%, respectively. Several compounds shared some of these prominent fragmentation peaks, such as *m*/*z* 105.0698 (corresponding to C_8_H_9_^+^) for compounds **3** and **4**; *m*/*z* 75.0444 (corresponding to C_3_H_7_O_2_^+^) for compounds **2**, **3**, and **6**; and *m*/*z* 71.0861 (C_5_H_11_^+^) for compounds **1** and **5**. In addition, all peaks for compound **7** were shared with those of compound **8**. In this small set of compounds, those that showed strong signals suggesting transesterification also showed strong protonated acetic acid peak at *m*/*z* 61 while the likely accompanying fragment at *m*/*z* 43 was not detected due to mass range limitations of the instrument. The most intense and most specific peaks are listed in Table 2. Oxidation via net loss of H_2_ was a common degradation path, observed in 50% of all compounds.

**Table 2:** Best and most intense *m*/*z* values of all eight compounds. The term ‘Best’ translates to the most intensive peak of high specificity (unique, not present in other compounds). Full table of all observed major *m*/*z* signals is shown in Table S4

No	Best *m*/*z*	Adduct	Most intense *m*/*z*	Adduct
1	131.1067	[M+H]^+^	71.0861	[M-C_2_H_4_O_2_+H]^+^
2	117.091	[M+H]^+^		
3	165.091	[M+H]^+^	105.0698	[M-C_2_H_4_O_2_+H]^+^
4	121.0647 123.0804	[M-2H+H]^+^ [M+H]^+^	105.0698	[M-H_2_O+H]^+^
5	103.0753	[M-2H+O+H]^+^		
6	89.0597	[M+H]^+^	61.0289	Acetate
7	153.1266	[M+H]^+^	135.1168	[M-H_2_O+H]^+^
8	157.1587	[M+H]^+^	95.0855	[M-C_3_H_10_O+H]^+^

We investigated the ionisation and in-source fragmentation behaviour for all compounds at three different dilutions (stock (5 μL/100 mL), 0.09-dilution, 0.0081-dilution). While changes in fragment ion ratios were observed, fragmentation remained extensive. The dimer formation in compounds **1** and **2** reduced significantly with lower concentrations; we have also observed a significant increase in the quasi-molecular ion for compound **3**, which increased from 0.28 to 133% of the fragment ion at *m*/*z* 105. We also see a relative increase of the protonated ion for compound **7** on the lowest concentration sample. However, negative influence towards the pseudo-molecular peak is observed for compound **2** for the loss of H_2_ (*m*/*z* 117 ➔ 115), compound **5** (*m*/*z* 89 ➔ 61), and fragmentation paths *m*/*z* 157 ➔ 95/137/173 for β-citronellol (**8**) (Table 3).

**Table 3:** Ratios of parent peak compared to the observed peaks at three different dilutions of pure standard. Only those compounds are included here that showed a quasi-molecular peak at [M+H]^+^ and ions not strongly affected by background signals

		Percent parent peak at dilution		
No	Mass ratio	1	0.09	0.081
1	131/261	222%	306%	12037%
2	117/115	132%	36%	-
	117/233	114%	196%	14335%
3	165/105	0.3%	0.2%	133%
7	153/169	506%	2231%	212%
	153/135	15%	34%	148%
	153/95	16%	15%	49%
8	157/173	157%	478%	210%
	157/137	191%	263%	24%
	157/95	36%	38%	18%

**Table 4:**
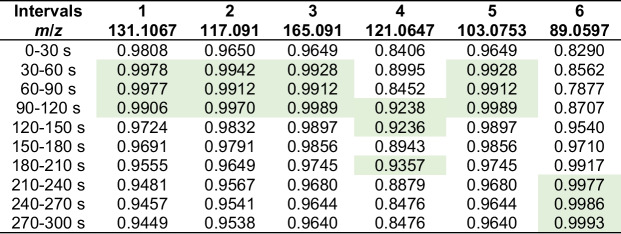
*R*^2^ values for linearity of the calibration curves in dependence of the different 30-s time intervals over the 5-min measurement duration. Highlighted in green are *R*^2^ values > 0.99 (or the best value)

A large proportion of ions observed from compounds **1**–**8** showed background interference through isobaric species. While higher concentration standard solutions showed orders of magnitude higher signal than the interferences, in case of the lowest analysed concentrations (2–4 μM range), signal was frequently indiscernible from background and thus limiting achievable detection limits. Particularly strong background was detected for ethyl acetate (**6**) and 2-phenyl ethanol (*m*/*z* 121.0647, **4**), both showing background signal in the range of >1E6 absolute intensity. Thus, we have tested the influence of an alternative atmosphere (Nitrogen N5.0) on the background contaminants relevant for this study, even though this reduces the ambient characteristics of DBDI. Comparison of spectra of both backgrounds is shown in Figure S3. While nitrogen-enriched atmosphere led to the appearance of other background peaks, overall, a significant reduction in background levels was observed for all relevant ions (see Table S5), with the majority of peaks reducing in intensity by more than a factor of 10. No reduction in formation of oxygenated species in compounds **7** and **8** were observed. We performed MS/MS of these background interferences and compared them to the MS/MS spectra of peaks observed in our standard solutions. We found a range, but not all of them, to be identical as it is summarised in Table S5.

### Comparison of different experimental setups

Methods A and C frequently led to the plasma extinguishing, either because the headspace was exhausted (method A) or the syringe content inserted too quickly (method C). A feasible injection speed in method C was 0.5 mL/min, which however, did not result in a good MS response for analysis. Resulting total ion chromatograms of these setups are shown in Figure S2. Good and sustained signal was observed for methods A and B while signal was not continuous and its appearance was highly compound dependent in method C. Method A led to short signal instabilities shortly after tube insertion. After sampling, sustained carry-over was observed for method A, especially for compounds **7**, **8**, and **4**, showing stable analyte levels post analysis for several minutes. No carry-over was observed for method B. Ambient sampling was found to yield the lowest standard deviations for all setups (see Table S6), ranging from 8 to 20% for the different compounds in standard mix 1.

### Analysis of the linearity of the data

**Fig. 2:**
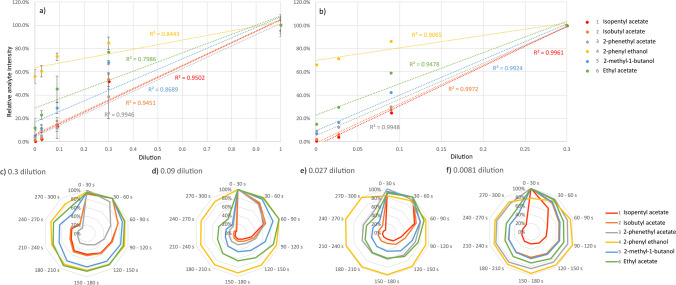
**a**, **b** Calibration curves starting at stock (5 μL/100 mL) and 0.3 dilution solution of standard mix 1, respectively. Curves were constructed from the integral over the whole 5-min (300 s) measurement duration. **c**–**f** Radar plots showing ion intensities over measurement duration in intervals of 30 s for concentrations of (**c**) 0.3, (**d**) 0.09, (**e**) 0.027, and (**f**) 0.081 dilution

Saturation effects were seen if the integral of the entire sampling duration of 5 min was formed for the highest concentration point for all standards, resulting in *R*^2^ values ranging from 0.7986 to 0.9766 (see Fig. 2a). If the highest concentration point is omitted, *R*^2^ values improved, ranging from 0.9478 to 0.9989 (see Fig. 2b); however, saturation was still observed in the case of compounds **4** and **6**. Linear ranges deducted from data shown in Fig. 2a, b are given in Table S2. While the upper limit of linearity is given by the highest linear datapoint, the lowest limit is determined by the lowest concentration tested (0.0081-dilution, 2–4 μM based on compound). Next, we assessed the signal stability and the linearity of the concentration curve over different 30 s time intervals of overall 5-min sample introduction duration. The radar plots in Fig. 2c–f show relative compound abundance over the 5-min sampling period divided into 30 s intervals for different standard dilutions. The highest signal intensities were obtained for all compounds in the earlier time ranges from 0 to 90 s. This effect is stronger for compounds **1** and **2** than for compounds **3**–**6**. Compound **4** stands out as it showed particularly stable signal intensities over a sustained period of time at all dilutions. Compounds **5** and **6** show a decrease in signal stability with time especially moving from 0.3- to 0.09-dilution. This coincides with a shift into the linear range for both compounds. Radar shapes displayed in Fig. 2f (dilution 0.0081) show a shift towards more sustained signal in case of several compounds due to the inability to distinguish these signals from isobaric background interferences. Comparing signal stability over time to compound properties such as boiling point, vapour pressure, and vapour density does not lead to any correlation with the observed behaviour.

The linearity of the calibration curves when plotted over data obtained from the different 30 s time intervals was assessed via the respective *R*^2^ values for the individual standard compounds. Consistently high *R*^2^ values > 0.95 over all time intervals were seen for all compounds except compounds **4** and **6** (Table 4). A dependence on the analysis time with specific optima however was observed for all compounds. While compounds **1**, **2**, **3**, and **5** were found to be most linear within the ranges between 30 and 120 s, compound **4** was found most linear in intermediate times of 90–210 s while compound **6** was found to behave most linearly in the later time points, improving significantly after 180 s. Notably, all compounds showed an increase in linearity from 0–30 s to 30–60 s windows for data extraction.

### Optimisation of the equilibration parameters

**Fig. 3:**
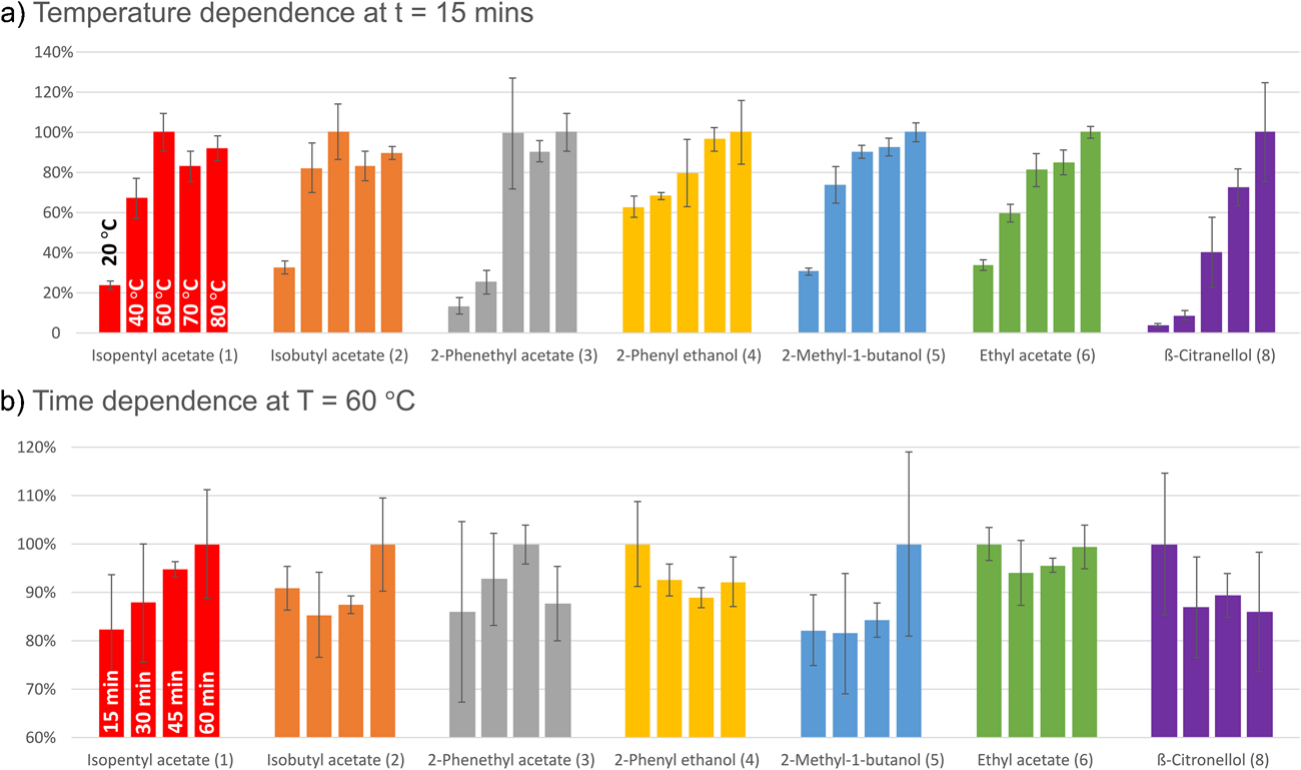
Dependence of signal intensity in 30–60-s interval with regard to equilibration time and temperature. **a** Temperature dependence at 20, 40, 60, 70, and 80 °C (at *t* = 15 min). **b** Time points: 15, 30, 45, 60 min (at *T* = 60 °C)

We have started the optimisation of the equilibration temperature with an equilibration duration of *t* = 30 min. A strong compound-specific effect was seen for equilibration temperature, with no single best temperature suitable for all compounds (see Figure S4a). While most compounds generally showed higher intensities at higher temperatures, several compounds showed an optimum at moderate temperatures, with further increase even leading to inferior intensities than were observed at room temperature (compounds **2**, **4**, **5**, and **6**). The highest summed relative intensities constituting the best compromise were found for 60 °C (see Figure S4b). Thus, we have next performed the determination of optimum extraction time at *T* = 60 °C (see Fig. 3a).

An improvement with equilibration time was seen especially for compound **1**, while the picture is more mixed for all other compounds (see Fig. 3b). Compounds **4**, **6**, and **8** behave similarly, preferring short equilibration times while compounds **2** and **5** preferred the longest time point. However, for all compounds and at all time points, relative ion intensity is above 80%. The summed relative ion intensities (from 0 to 100%) for all standards were indeed comparable for all time points, worst for 30 min, identical for 15 and 45 min, and best for 60 min (see Figure S4b). As intensities however were consistently high at all time points, we have chosen 15 min as best compromise between high throughput and sensitivity. The respective temperature dependence at *t* = 15 min can be found in Fig. 3a (summed ion intensities in Figure S4c). At *t* = 15 min, compounds **1** and **2** show maximum intensity at *T* = 60 °C while all other compounds apart from compound **8** lie above 80% of the maximum observed intensity.

### Analysis of fungal cultures

Due to these reasons, our final VOC screening method involved incubation at 60° for 15 min using direct introduction into the ion source at positive ion mode for 60 s. Data extraction for analysis is between 30 and 60 s analysis time only. We subsequently used this method to study 13 fungal cultures and 3 medium blanks from 3 different complex culturing media, potato medium with 4 g/L (medium A) or 20 g/L potato extract (medium B), as well as YPD with 10 mg/L yeast extract and 20 g/L peptone (medium C), all supplemented with 2% glucose.

**Fig. 4:**
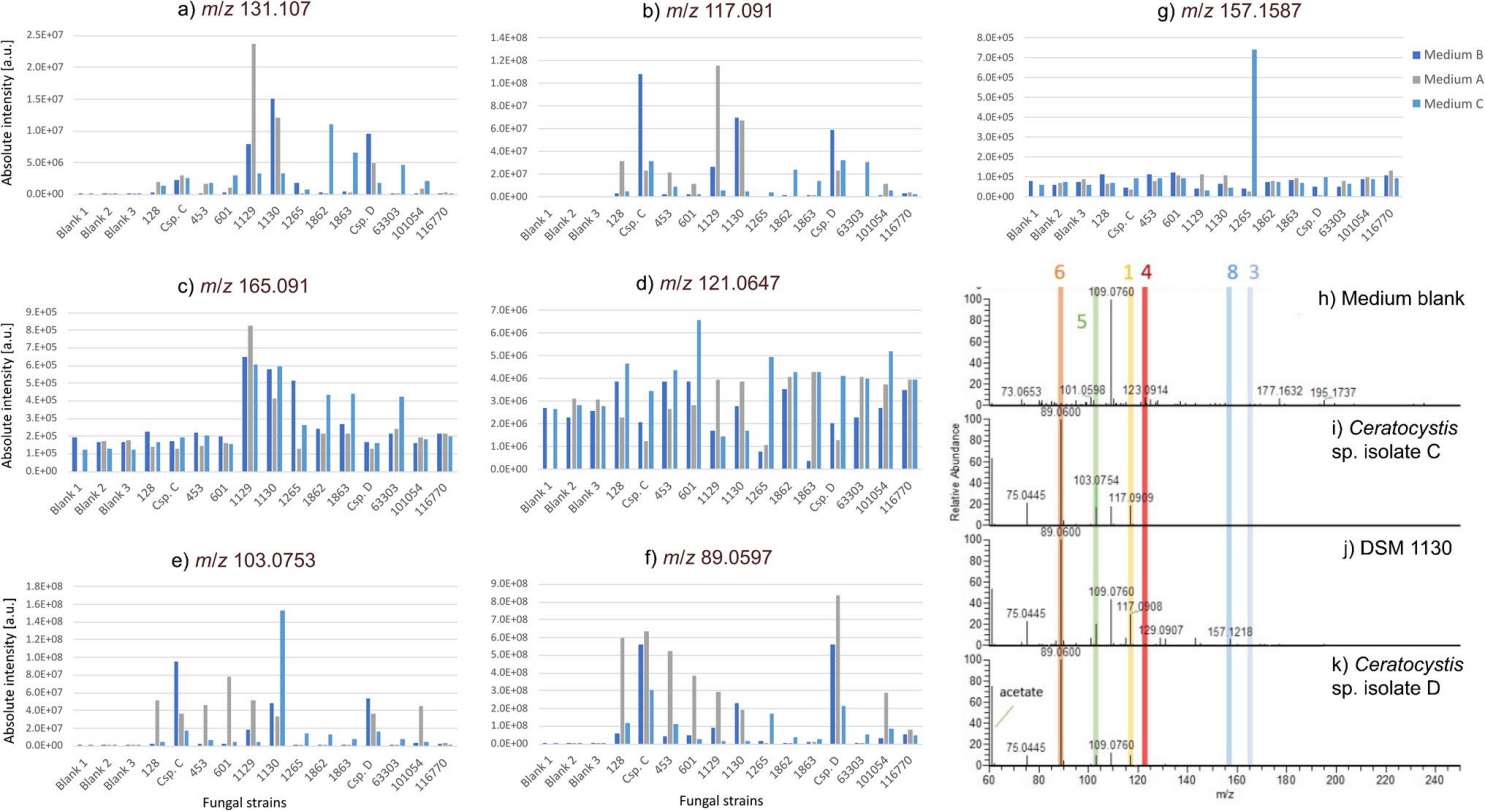
**a**–**g** Extracted intensity values for compounds of interest in 13 fungal cultures grown on three different media. Medium A: potato medium with 4 g/L of potato extract, medium B: potato with 20 g/L of potato extract, and medium C: YPD with 10 mg/L yeast extract and 20 g/L peptone; all media were supplemented with 2% glucose*.* **h**–**k** Positive ion mode spectra of selected fungal cultures and background/medium using SICRIT. (**h**) Medium B control, (**i**) *Ceratocystis* sp. isolate C, (**j**) DSM 1130, (**k**) *Ceratocystis* sp. isolate D. Highlighted are positions of compounds of **1**, **3**–**6**, and **8**

We assessed the production levels of the standard compounds within these 13 fungal strains using the ambient sampling approach. Production of aroma compounds was seen in all media, with best production media being compound and strain-specific (see Fig. 4a–g). Clear increases in fungal cultures vs medium background were observed for compounds **1**, **2**, **5**, and **6**. Less clear differences were found for compounds **3**, **4**, and **8** where changes between cultures and medium are smaller (<10-fold). Good producers for a compound could however still be clearly distinguished. The described workflow enables the choice of best strains and culturing media for each standard compound for optimisation of culturing conditions for most efficient aroma compound production. Strains DSM 1130, *Ceratocystis* sp. isolate C, and *Ceratocystis* sp. isolate D were efficient aroma compound producers for compounds **1**, **2**, **5**, and **6** (see Fig. 4h–k) compounds which are frequently produced together by individual strains. The same strains, especially *Ceratocystis* sp. isolates C and D, are not producing compounds **8** and **3**.

The analysis in all three different media was performed twice, on different days and aliquots of the same cultures to assess analytical reproducibility. A good correlation was observed between both analytical replicates, with *R*^2^ values ranging from 0.6559 to 0.919 (see Figure S5). It was observed that among all three culturing media, data of samples grown in medium B showed a larger spread than those grown in media A and C. Correlations with *R*^2^ values > 0.8 were consistently observed for compounds that spanned several orders of magnitude in intensity (compounds **1**, **2**, **5**, **6**, **8**) while values below 0.8 were observed for those compounds spanning a single order of magnitude only (compounds **3**, **4**). This suggests that while the method is less sensitive in distinguishing small intensity differences, it is reliably and quickly detecting larger differences in production levels.

## Discussion

All compounds tested during this study could be ionised with DBDI, with positive ion mode superior for detection in all cases*.* However, the observed isobaric background and complex ionisation behaviour including extensive fragmentation on even this comparatively small pool of VOCs might potentially seriously restrict the application of the SICRIT technology for direct, untargeted VOC profiling using a Thermo Q-Exactive instrument. Compound **7** for instance cannot be determined in presence of compound **8** as it shares all fragments with compound **8**. It requires knowledge of the spectral appearance of each compound in isolation in order to determine the most abundant and the most specific peaks, which frequently are not the same *m*/*z* species. This is particularly clear in cases of compounds **3**, **4**, **5**, **7**, and **8**, for which the base peak is in most cases a smaller mass weight fragment or in the case of compound **5** an oxidation product. Further complicating this matter is the fact that many fragment peaks, even in this small pool of aroma compounds, were not specific. This results in a need to know all fragmentation peaks and possible source compounds before embarking on untargeted analysis. Observed fragments and adducts were produced in a concentration-dependent manner; however, this did not seem to interfere with the detection of the peak best suited for identification as listed in Table 2. In addition, transesterification was observed in the form a signal at *m*/*z* 75 which was ascribed to a transesterification product in which the ethyl- and isobutyl-residue of compounds **6** and **2**, respectively, is replaced with a methyl-group. Although transesterifications are usually catalysed through strong acid or bases, we have analysed the same compounds in pure aqueous solution and found the signal for the methyl acetate (*m*/*z* 75) only in those samples that contained added methanol (see Figure S6). Alcoholic solutions are thus better avoided if esters are to be analysed using DBDI. In general, we have observed that functional groups such as aliphatic hydroxyls and carbonyls seem prone to water loss as well as addition of oxygen (often under loss of H_2_). Detection limits were in the lower μM range for all compounds, largely determined by high levels of isobaric background interferences. The use of a controlled background atmosphere (like nitrogen or synthetic air) is expected to lead to a reduction of background interferences and thus detection limits by at least an order of magnitude for most compounds.

We have tested three different experimental procedures for introduction of the headspace into the DBDI ion source, using either a tube (A), a syringe (C), or ambient sampling (B). Method B was chosen to be developed further during this study as it exhibited the best reproducibility among all setups tested, lowest carry-over, and the highest degree of simplicity. Ambient sampling was the only method that swiftly returned to background levels after analysis, a feature that is important to enable high sample throughout. Extended carry-over, especially for compounds with high boiling points (above 200 °C), was seen in any setup involving even just short transfer lines of polymer or metal. This phenomenon might be overcome by using heated transfer lines; however, building a fully optimised heated setup with controlled background atmosphere was beyond the scope of the current study which was aimed at testing DBDI under simplified conditions for the high-throughput, first-tier screening of fungal aroma compounds. Using this setup, direct analysis by DBDI using the SICRIT source proved capable of analysing aroma compounds in a very simple and fast manner. With the exception of the highest concentration points (5 μL/100 mL), data was found largely linear, enabling comparison of relative abundances between samples for qualitative analysis. Initially, sampling was performed over a 5 min period to investigate how stable the signal is over time and whether this effect is concentration dependent. All compounds at all dilutions showed highest signal intensities in the range of 0–90 s before signal decreased over the remaining time course (with the notable exception of compound **4** which remained at stable levels over the entire time period tested). We tested the linearity of the data in 30 s intervals and found the best results for the majority of the compounds for the time period between 30-60 s. Thus, in order to generate a short analysis method, data analysis for 1 min followed by data extraction in the interval of 30–60 s was chosen for data extraction in further experiments.

Although the final overall method duration was 5 min (1 min background, 1 min ambient sampling, 2 min background), an overall shortened method of 2 min/sample seems to be feasible due to the absence of carry-over using the ambient sampling setup. Stationary equilibration of as little as 15 min at 60 °C was sufficient to achieve good signal intensities for all standards. Compounds with lower boiling points (**1**, **2**, **5**, **6**) generally show less temperature dependence than those compounds with high boiling points (> 200 °C). Although several compounds showed highest signal intensities at *t* = 15 min at the highest temperatures used for equilibration (*T* = 80 °C), some showed a decrease in intensity beyond *T* = 60 °C, possibly suggesting thermal breakdown of compounds. Due to the unknown and heterogenous nature of the fungal samples being studied and the overall aim being the development of a rapid ultimately untargeted screening method, we have decided that for us 60 °C at *t* = 15 min represents the best and most widely applicable compromise in equilibration conditions. However, in order to obtain optimum conditions for a targeted analyte, equilibration temperature and time have to be optimised including temperatures going beyond 80 °C.

Having determined that we can gain qualitative information and are working within the linear range of the compounds with our methods tested, we have subsequently deployed it to analyse samples of 13 fungal strains in three different culturing media for the medium with the best production levels of aroma compounds. The only sample preparation performed was a filtering to remove the fungal mycel. As such, the analysed sample contains the media base as well as all secreted fungal natural products. Supported by our optimisation of the equilibration phase, we opted to keep the incubation temperature at moderate temperatures and times to bring as much VOCs as possible to the gas phase while at the same time not damaging temperature-sensitive compounds. Literature reports have used temperatures as low as 40 °C; this might however decrease the detection of low-level compounds. As the aim of the current study was to develop a fast aroma compound screening method, we have not normalised our data to the amount of fungal biomass. Instead, all fungal strains were cultured under identical conditions. The growth morphology for the tested fungal species is different based on the strain within the same medium, and so is their biomass and natural product secretion. In addition, production levels of aroma compounds might not be linearly related to fungal biomass. DBDI offers a feasible solution to determine the optimum culturing conditions for the amount of volatile production in a given liquid volume. Thus, DBDI could be a valuable tool to determine these complex relationships of culturing conditions, fungal biomass, and aroma compound production in a high-throughput fashion as compared to the standard GC-MS analysis. Once promising culturing conditions are identified, these can be subjected to second tier, more detailed characterisation using GC-MS.

We have analysed the fungal cultures in independent triplicate runs and found good agreement between relative abundances of the different replicates of the same samples. Better correlation was observed for such samples that span more than one order of magnitude in intensity values, indicating good sensitivity to distinguish large differences in production levels whereas small differences are distinguished with less sensitivity. The latter is the case with *m*/*z* 165 and *m*/*z* 123, both of which are low relative abundance fragments and close to atmospheric background interferences.

The sample draw for the SICRIT method, 18 mL gas phase, is much higher than the one available for GC-MS measurement with 0.5 mL injection volume. Therefore, we expect DBDI to be able to detect low-intensity VOCs, which might not be visible with the common GC-MS method at the same sample volume and treatment temperature. LODs are currently restricted due to high background interferences; however, a preliminary experiment deploying a Nitrogen N5.0 atmosphere suggests that for most compounds, an increase by an order of magnitude or more can be achieved using a purified atmosphere. However, more analysis and method development needs to be performed to transform this method into an untargeted screening method by identifying more fungal aroma compounds and characterising their ionisation behaviour as we have observed frequent fragmentation among our studied analytes.

## Conclusion

We tested a dielectric barrier discharge plasma source connected to an Orbitrap mass spectrometer with direct sampling as a novel method for the rapid analysis and screening of volatile aroma compounds in the headspace of fungal samples. This method showed good reproducibility with the limit of detection being in the lower μM range for all compounds. DBDI is shown to be a potentially useful tool in the large-scale screening of culturing conditions to achieve maximum volatile production in a fixed liquid volume. For our compounds of interest, we have found isobaric species detected in the background and observed complex ionisation and adduct formation behaviour in the ion source. While these points make this method currently unsuitable for the untargeted analysis of the VOCs found in the fungal headspace, a recent publication described that changes to the atmosphere have been shown to affect ionisation behaviour [57] to some extent. However, clear differences in the aroma compound production among our studied cultures were clearly visible and reproducible, indicating that with more database building to characterise spectral profiles of aroma compounds of interest, this method might have a place in biotechnological laboratories as high-throughput and easy-to-use method for culture condition optimisation.

## Supplementary Information

ESM 1 (PDF 817 kb)

## References

[1] Chambergo FS, Valencia EY (2016). Fungal biodiversity to biotechnology. Appl Microbiol Biotechnol..

[2] Wongsuk T, Pumeesat P, Luplertlop N (2016). Fungal quorum sensing molecules: role in fungal morphogenesis and pathogenicity: quorum sensing in fungi. J Basic Microbiol..

[3] Dixon EF, Hall RA (2015). Noisy neighbourhoods: quorum sensing in fungal-polymicrobial infections: quorum sensing in fungal infections. Cell Microbiol..

[4] Minerdi D, Maggini V, Fani R (2021). Volatile organic compounds: from figurants to leading actors in fungal symbiosis. FEMS Microbiol Ecol..

[5] Jalinas J, Lopez-Moya F, Marhuenda-Egea FC, Lopez-Llorca LV (2022). Beauveria bassiana (Hypocreales: Clavicipitaceae) volatile organic compounds (VOCs) repel Rhynchophorus ferrugineus (Coleoptera: Dryophthoridae). J Fungi..

[6] Wang Y, Lim L, Madilao L, Lah L, Bohlmann J, Breuil C (2014). Gene discovery for enzymes involved in limonene modification or utilization by the mountain pine beetle-associated pathogen Grosmannia clavigera. Cullen D, editor. Appl Environ Microbiol..

[7] Cale JA, Ding R, Wang F, Rajabzadeh R, Erbilgin N (2019). Ophiostomatoid fungi can emit the bark beetle pheromone verbenone and other semiochemicals in media amended with various pine chemicals and beetle-released compounds. Fungal Ecol..

[8] Asensio D, Rapparini F, Peñuelas J (2012). AM fungi root colonization increases the production of essential isoprenoids vs. nonessential isoprenoids especially under drought stress conditions or after jasmonic acid application. Phytochemistry..

[9] Li N, Alfiky A, Vaughan MM, Kang S (2016). Stop and smell the fungi: fungal volatile metabolites are overlooked signals involved in fungal interaction with plants. Fungal Biol Rev..

[10] Alfiky A, Weisskopf L (2021). Deciphering Trichoderma–plant–pathogen interactions for better development of biocontrol applications. J Fungi..

[11] Contreras-Cornejo HA, Orozco-Granados O, Ramírez-Ordorica A, García-Juárez P, López-Bucio J, Macías-Rodríguez L (2022). Light and mycelial injury influences the volatile and non-volatile metabolites and the biocontrol properties of Trichoderma atroviride. Rhizosphere..

[12] Poveda J (2021). Beneficial effects of microbial volatile organic compounds (MVOCs) in plants. Appl Soil Ecol..

[13] McNeal KS, Herbert BE (2009). Volatile organic metabolites as indicators of soil microbial activity and community composition shifts. Soil Sci Soc Am J..

[14] Frey-Klett P, Burlinson P, Deveau A, Barret M, Tarkka M, Sarniguet A (2011). Bacterial-fungal interactions: hyphens between agricultural, clinical, environmental, and food microbiologists. Microbiol Mol Biol Rev..

[15] Cugini C, Calfee MW, Farrow JM, Morales DK, Pesci EC, Hogan DA (2007). Farnesol, a common sesquiterpene, inhibits PQS production in Pseudomonas aeruginosa. Mol Microbiol..

[16] Fraatz MA, Zorn H. Fungal flavours. In: Hofrichter M, editor. Industrial applications [Internet]. Berlin, Heidelberg: Springer Berlin Heidelberg; 2011 [cited 2022 Dec 15]. p. 249–68. Available from: http://link.springer.com/10.1007/978-3-642-11458-8_12

[17] Panakkal EJ, Kitiborwornkul N, Sriariyanun M, Ratanapoompinyo J, Yasurin P, Asavasanti S, et al. Production of food flavouring agents by enzymatic reaction and microbial fermentation. Appl Sci Eng Prog [Internet]. 2021 Apr 29 [cited 2022 Dec 15]; Available from: http://ojs.kmutnb.ac.th/index.php/ijst/article/view/5012

[18] Kumar Verma D, Thyab Gddoa Al-Sahlany S, Kareem Niamah A, Thakur M, Shah N, Singh S (2022). Recent trends in microbial flavour Compounds: a review on Chemistry, synthesis mechanism and their application in food. Saudi. J Biol Sci..

[19] EFSA Panel on Additives and Products or Substances used in Animal Feed (FEEDAP), Bampidis V, Azimonti G, de Bastos M L, Christensen H, Dusemund B, *et al.* Safety of 37 feed additives consisting of flavouring compounds belonging to different chemical groups for use in all animal species (FEFANA asbl). EFSA J [Internet]. 2022 Apr [cited 2022 Dec 15];20(4). Available from: https://data.europa.eu/doi/10.2903/j.efsa.2022.7249 10.2903/j.efsa.2022.7249 PMC9016716 35464872

[20] Silva BAN, Eskinazi S, Jacob DV, Araujo WAG, Rebordões FIG, Gonçalves MF (2021). Feed flavour supplementation improves kinetics of intake and feeding behaviour pattern of lactating sows in a tropical climate. Livest Sci..

[21] Maurya R, Patel H, Bhatt D, Shakhreliya S, Gohil N, Bhattacharjee G, *et al.* Microbial production of natural flavors and fragrances. In: Kumar A, Patruni K, Singh V, editors. Recent advances in food biotechnology [Internet]. Singapore: Springer Nature Singapore; 2022 [cited 2022 Dec 15]. p. 139–59. Available from: https://link.springer.com/10.1007/978-981-16-8125-7_7

[22] Bicas JL, Neri-Numa IA, Ruiz ALTG, De Carvalho JE, Pastore GM (2011). Evaluation of the antioxidant and antiproliferative potential of bioflavors. Food Chem Toxicol..

[23] Food Flavors Market Size, Share & Trends | Report Analysis, 2030 [Internet]. Allied Market Research. [cited 2022 Dec 15]. Available from: https://www.alliedmarketresearch.com/food-flavors-market

[24] de Oliveira Felipe L, de Oliveira AM, Bicas JL (2017). Bioaromas – perspectives for sustainable development. Trends Food Sci Technol..

[25] Kemper N, Rainey R, Rainey D. Consumer preferences and the market for non-genetically modified food. 2014;

[26] Krause J, Tobi G. Discovery, development, and regulation of natural products. In: Kulka M, editor. Using old solutions to new problems - natural drug discovery in the 21st century [Internet]. InTech; 2013 [cited 2022 Dec 15]. Available from: http://www.intechopen.com/books/using-old-solutions-to-new-problems-natural-drug-discovery-in-the-21st-century/discovery-development-and-regulation-of-natural-products

[27] Bier MCJ, Medeiros ABP, De Kimpe N, Soccol CR (2019). Evaluation of antioxidant activity of the fermented product from the biotransformation of R-(+)-limonene in solid-state fermentation of orange waste by Diaporthe sp. Biotechnol Res Innov..

[28] de Souza Sevalho E, Paulino BN, de Souza AQ, de Souza AD Fungal biotransformation of limonene and pinene as a biotechnological approach for production of aroma compounds [Internet]. In Review; 2021 Jun [cited 2022 Dec 15]. Available from: https://www.researchsquare.com/article/rs-447822/v1

[29] Guo J, Zhang M, Fang Z (2022). Valorization of mushroom by-products: a review. J Sci Food Agric..

[30] Bennett JW, Hung R, Lee S, Padhi S. 18 fungal and bacterial volatile organic compounds: an overview and their role as ecological signaling agents. In: Hock B, editor. Fungal associations [Internet]. Berlin, Heidelberg: Springer Berlin Heidelberg; 2012 [cited 2022 Dec 15]. p. 373–93. Available from: http://link.springer.com/10.1007/978-3-642-30826-0_18

[31] Bäck J, Aaltonen H, Hellén H, Kajos MK, Patokoski J, Taipale R (2010). Variable emissions of microbial volatile organic compounds (MVOCs) from root-associated fungi isolated from Scots pine. Atmos Environ..

[32] Müller A, Faubert P, Hagen M, Zu Castell W, Polle A, Schnitzler JP (2013). Volatile profiles of fungi – chemotyping of species and ecological functions. Fungal Genet Biol..

[33] Gong D, Bi Y, Zong Y, Li Y, Sionov E, Prusky D (2022). Characterization and sources of volatile organic compounds produced by postharvest pathogenic fungi colonized fruit. Postharvest Biol Technol..

[34] Brilli F, Luchi N, Michelozzi M, Calamai L, Cencetti G, Pecori F (2020). Volatile organic compounds (VOC) as biomarkers for detection of *Ceratocystis platani*. For Pathol..

[35] Karsli A, Şahin YS (2021). The role of fungal volatile organic compounds (FVOCs) in biological control. Türkiye Biyolojik Mücadele Derg..

[36] Lanza E, Ko KH, Palmer JK (1976). Aroma production by cultures of Ceratocystis moniliformis. J Agric Food Chem..

[37] Rottava I, Toniazzo G, Cortina PF, Martello E, Grando CE, Lerin LA (2010). Screening of microorganisms for bioconversion of (−)β-pinene and R-(+)-limonene to α-terpineol. LWT - Food Sci Technol..

[38] Rottava I, Cortina PF, Martello E, Cansian RL, Toniazzo G, Antunes OAC (2011). Optimization of α-terpineol production by the biotransformation of R-(+)-limonene and (−)-β-pinene. Appl Biochem Biotechnol..

[39] Li M, Xiao Y, Zhong K, Wu Y, Gao H (2022). Delving into the biotransformation characteristics and mechanism of steamed green tea fermented by Aspergillus niger PW-2 based on metabolomic and proteomic approaches. Foods..

[40] Pastore GM, Park YK, Min DB (1994). Production of fruity aroma by Neurospora from beiju. Mycol Res..

[41] Tatum EL, Gross SR, Ehrensvärd G, Garnjobst L (1954). Synthesis of aromatic compounds by Neurospora. Proc Natl Acad Sci..

[42] de Carvalho DS, Dionísio AP, dos Santos R, Boguzs S, Godoy HT, Pastore GM (2011). Production of 1-octen-3-ol by Neurospora species isolated from beiju in different culture medium. Procedia Food Sci..

[43] Na N, Zhao M, Zhang S, Yang C, Zhang X (2007). Development of a dielectric barrier discharge ion source for ambient mass spectrometry. J Am Soc Mass Spectrom..

[44] Weber M, Wolf JC, Haisch C (2021). Gas chromatography–atmospheric pressure inlet–mass spectrometer utilizing plasma-based soft ionization for the analysis of saturated, aliphatic hydrocarbons. J Am Soc Mass Spectrom..

[45] Adamovich I, Baalrud SD, Bogaerts A, Bruggeman PJ, Cappelli M, Colombo V (2017). The 2017 plasma roadmap: low temperature plasma science and technology. J Phys Appl Phys..

[46] Gyr L, Klute FD, Franzke J, Zenobi R (2019). Characterization of a nitrogen-based dielectric barrier discharge ionization source for mass spectrometry reveals factors important for soft ionization. Anal Chem..

[47] Mirabelli MF, Wolf JC, Zenobi R (2017). Atmospheric pressure soft ionization for gas chromatography with dielectric barrier discharge ionization-mass spectrometry (GC-DBDI-MS). The Analyst..

[48] Elke K, Begerow J, Oppermann H, Kråmer U, Jermann E, Dunemann L (1999). Determination of selected microbial volatile organic compounds by diffusive sampling and dual-column capillary GC-FID-a new feasible approach for the detection of an exposure to indoor mould fungi?. J Environ Monit..

[49] Wolf JC, Schaer M, Siegenthaler P, Zenobi R (2015). Direct quantification of chemical warfare agents and related compounds at low ppt levels: comparing active capillary dielectric barrier discharge plasma ionization and secondary electrospray ionization mass spectrometry. Anal Chem..

[50] Thaler KM, Gilardi L, Weber M, Vohburger A, Toumasatos Z, Kontses A (2021). HELIOS/SICRIT/mass spectrometry for analysis of aerosols in engine exhaust. Aerosol Sci Technol..

[51] Liu Y, Lin Z, Zhang S, Yang C, Zhang X (2009). Rapid screening of active ingredients in drugs by mass spectrometry with low-temperature plasma probe. Anal Bioanal Chem..

[52] Campbell DI, Dalgleish JK, Cotte-Rodriguez I, Maeno S, Graham CR (2013). Chemical analysis and chemical imaging of fragrances and volatile compounds by low-temperature plasma ionization mass spectrometry: analysis of volatiles and fragrances by LTP/MS. Rapid Commun Mass Spectrom..

[53] Bluemke W, Schrader J (2001). Integrated bioprocess for enhanced production of natural flavors and fragrances by Ceratocystis moniliformis. Biomol Eng..

[54] Elia EA, Niehaus M, Steven RT, Wolf JC, Bunch J (2020). Atmospheric pressure MALDI mass spectrometry imaging using in-line plasma induced postionization. Anal Chem..

[55] Michael JA, Mutuku SM, Ucur B, Sarretto T, Maccarone AT, Niehaus M, *et al.* Mass spectrometry imaging of lipids using MALDI coupled with plasma-based post-ionization on a trapped ion mobility mass spectrometer. Anal Chem. 2022;acs.analchem.2c03745. 10.1021/acs.analchem.2c03745 36473074

[56] Funke SKI, Brückel VA, Weber M, Lützen E, Wolf JC, Haisch C (2021). Plug-and-play laser ablation-mass spectrometry for molecular imaging by means of dielectric barrier discharge ionization. Anal Chim Acta..

[57] Weber M, Wolf JC, Haisch C (2023). Effect of dopants and gas-phase composition on ionization behavior and efficiency in dielectric barrier discharge ionization. J Am Soc Mass Spectrom..

