# Supplementary material for: Screening of volatile organic compounds (VOCs) from liquid fungal cultures using ambient mass spectrometry
Source: Anal Bioanal Chem. 2023 Jun 30;415(18):4615–27. doi: 10.1007/s00216-023-04769-6 (PMC10329071; doi:10.1007/s00216-023-04769-6)
Supplement: Supplementary file 1 — (PDF 817 kb) [file 216_2023_4769_MOESM1_ESM.pdf]

SUPPLEMENTARY INFORMATION

Screening of volatile organic compounds (VOCs) from liquid fungi cultures using ambient mass spectrometry

Daniel Heffernan[1,T], Melania Pilz[2,T], Marco Klein[1], Martina Haack[2], Alan M. Race[3], Thomas Brück[2], Farah Qoura[2], Nicole Strittmatter[1,*]

1 Department of Biosciences, TUM School of Natural Sciences, Technical University of Munich (TUM), Garching, Germany

2 Department of Chemistry, TUM School of Natural Sciences, Technical University of Munich (TUM), Garching, Germany

3 Institute of Medical Bioinformatics and Biostatistics, University of Marburg, Germany

[T]authors contributed equally.

*Corresponding author: nicole.strittmatter@tum.de

Content: Table comprising literature review of previously characterised aroma compounds in related fungal strains, concentration values corresponding to dilution factors deployed in main study, list of all fungal strains used, comparison of spectra to deduct source of transesterification, table of all adducts and fragments observed in this study for compounds **1-8**, characterisation of different experimental setups and sampling atmospheres on spectral appearance and measurement profiles, summed relative ion intensities for different equilibration temperatures and lengths, VOC production profiles for 13 different fungal strains in three complex media, correlation plots of screening data obtained for three replicate measurements.

Table S1. Flavour profiles of a selected variety of *Ceratocystis* sp., *Aspergillus* sp. and *Neurospora* sp., associated media compositions and analysis methods for fungal aroma profiles of relevant publications.

Strain	Medium	Aroma	Carbon source	Nitrogen source	Analysis method	VOC	Source
Ceratocystis moliniformis	basal medium according to Wilson and Lilly, 1958	fruity, banana	dextrose 3 %	Urea leucine isoleucine norleucine	Headspace GC/MS	ethanol, ethyl acetate, isoamyl acetate, geranial, citronellol, γ- and δ-decalactone, n-propyl acetate, isobutyl acetate, amyl acetate (2-methyl butyl acetate)	Lanza *et al*., 1976[1]
		fruity, grapefruit, lemon	dextrose 3 %	glycine			
		canned peach, pear	glycerol	urea 0.1 %			
		tropical banana, cantaloupe	corn starch	urea 0.1 %			
Ceratocystis moliniformis	potato-dextrose broth, basal medium according to Wilson and Lilly, 1958 modified	fruity, banana, citrus, floral	dextrose 3 %	0.38 gL^{-1} urea	GC	ethyl acetate, propyl acetate, isobutyl acetate, isoamyl acetate, citronellol, geraniol	Bluemke *et al*., 2001[2]
Aspergillus niger	potato-dextrose broth 4 gL^{-1}, supplementation: (−)-β-pinene and R-(+)-limonene	floral, citrus, pine-wood, herbaceous, camphor	glucose 2 %	-	GC/MS	α-terpineol, trans-pinocarveol, pinocamphone, fenchol	Rottava *et al*., 2010[3]; Rottava *et al*., 2011[4]
Aspergillus niger PW-2	720 g sterilized green tea leaves + 270 mL fungal culture	green tea flavour profile	-	-	HS-SPME/GC-MS	linalool, 1-octen-3-ol, geraniol, L-α-terpineol, (E)-linalool oxide, benzaldehyde, nonanal, ethyl palmitate, (Z)-geranylacetone, 2,4-di-tert-butylphenol (excerpt)	Li *et al*., 2022[5]
Neurospora sp.	Czapeck modified medium	mushroom aroma	Sucrose 30 gL^{-1}	$NH_4H_2PO_4$ 40.6 gL^{-1}	GC/MS	1-octen-3-ol	De Carvalho *et al*., 2011[6]

	malt extract broth 50 gL^{-1}						
	yeast malt broth 5 gL^{-1} peptone, 3 gL^{-1} yeast extract, 3 gL^{-1} malt extract		glucose 10 gL^{-1}	-			
	yeast extract 5 gL^{-1}		fructose 50 gL^{-1}	-			
Neurospora sp.	5% malt extract medium	fruity, mushroom	-	-	Headspace GC/MS	ethyl hexanoate, 3-methyl-1-butanol, 1-octen-3-ol, ethyl acetate, ethanol	Pastore *et al.*, 1994[7]

Table S2. Concentration values [mol/L] for all standards used in this study.

		concentration at dilution factor [mol/L]					Linear range
**Name**	**cmpd**	**1**	**0.3**	**0.09**	**0.027**	**0.0081**	**(dilution factor)**
isopentyl acetate	**1**	3.36E-04	1.01E-04	3.03E-05	9.08E-06	2.73E-06	0.3-0.0081
isobutyl acetate	**2**	3.77E-04	1.13E-04	3.39E-05	1.02E-05	3.05E-06	0.3-0.0081
2-phenethyl acetate	**3**	3.31E-04	9.94E-05	2.98E-05	8.95E-06	2.68E-06	1-0.0081
2-phenyl ethanol	**4**	4.17E-04	1.25E-04	3.76E-05	1.13E-05	3.38E-06	0.09-0.0081
2-methyl-1-butanol	**5**	4.63E-04	1.39E-04	4.17E-05	1.25E-05	3.75E-06	0.3-0.0081
ethyl acetate	**6**	5.12E-04	1.54E-04	4.61E-05	1.38E-05	4.15E-06	0.09-0.0081
citral	**7**	2.91E-04	8.74E-05	2.62E-05	7.86E-06	2.36E-06	
β-citronellol	**8**	2.74E-04	8.23E-05	2.47E-05	7.40E-06	2.22E-06	

Table S3. *Ascomycete* fungal strains chosen for the method development.

**Strain**	**Identifier**
Ceratocystis sp. isolate C	Csp C
Ceratocystis sp. isolate D	Csp D
Ceratocystis paradoxa	CBS 101054
Ceratocystis paradoxa	CBS 601.70
Ceratocystis paradoxa	CBS 128.32
Ceratocystis paradoxa	CBS 453.66
Ceratocystis paradoxa	CBS 116770
Aspergilus oryzae	DSM 1862
Aspergilus oryzae	DSM 1863
Aspergilus oryzae	DSM 63303
Neurospora crassa	DSM 1129
Neurospora sitophila	DSM 1130
Neurospora intermedia	DSM 1265

Table S4. Table detailing ions observed for the 8 investigated compounds using SICRIT.

**Number**<br>**Name**	**1**<br>**Isopentyl acetate**	**2**<br>**Isobutyl acetate**	**3**<br>**2-phenethyl acetate**	**4**<br>**2-phenyl ethanol**	**5**<br>**2-methyl-1-butanol**	**6**<br>**Ethyl acetate**	**7**<br>**citral**	**8**<br>**β-citronellol**
	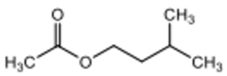	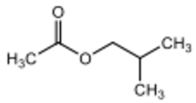	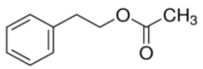	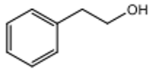	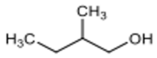	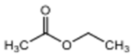	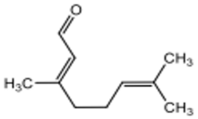	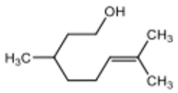
Sum formula	C7H14O2	C6H12O2	C10H12O2		C5H12O	C4H8O2	C10H16O	C10H20O
$[M+H]^+$	131.106656	117.091006	165.091006	123.080441	-	89.05971	153.127391	157.158691
$[M-H_2O+H]^+$				105.069876	71.085526		135.116826	139.148126
$[2M+H]^+$	261.206036	233.174736			177.184906			
$[M-H_2+H]^+$	129.0908	115.0753	163.0745	121.0647	87.0806		151.111	155.1422
$[M-C_2H_4O_2+H]^+$	71.0861		105.0698					
$[M-H_2+O+H]^+$			179.0694	137.0591	103.0753			171.137
$[C_3H_7O_2]^+$		75.0444	75.0444	75.0444		75.0444		
$[C_2H_5O_2]^+$		61.0289				61.0289		
$[M-C_2H_6O+H]^+$							107.0853	
$[M-C_3H_6O+H]^+$			95.0856				95.0855	
$[M-C_3H_8O+H]^+$							93.07	
$[M-C_4H_8O+H]^+$							81.0701	
$[M+O+H]^+$							169.1214	173.1527
$[M-H_4+H]^+$					85.0651			153.1267
$[M-H_4+O+H]^+$					101.0599			169.1214
$[M-C_3H_{10}O+H]^+$								95.0855
$[M-C_3H_8+H]^+$								113.1318
$[M-C_4H_{12}O+H]^+$								81.0701
$[M-C_4H_{10}O+H]^+$								83.0859

Base peak is highlighted in green, while second and third highest peaks are indicated in orange and red, respectively. Grey font indicates that abundance compared to base peak is less than 5%.

Table S5. *m/z* values observed for compounds **1-8** and their background intensities under ambient and nitrogen rich atmospheres. MS/MS spectra were recorded for all present ions and compared to those recorded from the standard solutions. If these were visually highly similar with regards to ratios and presence/absence of peaks, identity was regarded as confirmed.

m/z value	ambient	Nitrogen N5.0	-fold decrease	Same as standard
61.0289	2.47E+06	4.15E+05	6.0	n
71.0861	6.90E+05	2.81E+04	24.6	n
75.0444	5.28E+05	7.19E+04	7.3	y
81.0705	2.39E+06	3.14E+04	76.1	y
87.0809	6.86E+05	1.90E+04	36.1	y
89.0597	1.59E+06	3.98E+04	39.9	n
89.0961	-	-	-	
95.0856	9.92E+05	3.49E+04	28.4	y
103.0756	1.80E+05	1.54E+04	11.7	y
105.0698	8.05E+05	1.49E+05	5.4	y
107.0857	5.13E+05	6.20E+03	82.7	y
115.0753	1.09E+06	2.51E+04	43.4	y
117.0910	2.59E+05	1.02E+04	25.4	n
121.0648	4.67E+06	3.10E+04	150.6	y
129.0908	4.93E+05	1.47E+04	33.5	n
131.1067	7.71E+04	5.49E+03	14.0	n
135.1168	3.24E+05	6.98E+03	46.4	y
137.0595	2.48E+05	1.23E+04	20.2	y
153.1271	6.43E+05	9.52E+03	67.5	y
163.0785	1.34E+05	9.40E+04	1.4	n
165.0907	1.18E+05	3.44E+03	34.3	n
169.1220	3.14E+05	6.83E+03	46.0	y
179.0699	1.94E+04	2.45E+03	7.9	n

Table S6. Reproducibility and signal strength of the three developed methods. The reproducibility is measured by the standard deviation, and the signal strength by the mean values of the integrals. Method A is shown to have the highest signal strength, but poor robustness. Method C has low signal strength and low reproducibility. Method B is shown to have the best signal strength and reproducibility.

**Method A**		**Average Integral**	**Standard Deviation [%]**
	Overall measurement	4.68E+11	15.51
	Isopentyl acetate	6.45E+08	36.79
	2-Phenylethyl acetate	3.09E+11	16.4
	Ethyl acetate	1.01E+09	42.61
**Method B**		**Average Integral**	**Standard Deviation [%]**
	Overall measurement	3.52E+11	12.01
	Isopentyl acetate	2.58E+10	13.54
	2-Phenylethyl acetate	1.54E+11	21.22
	Ethyl acetate	6.35E+09	8.1
**Method C**		**Average Integral**	**Standard Deviation [%]**
	Overall measurement	2.36E+09	14.8
	Isopentyl acetate	5.21E+06	47.97
	2-Phenylethylacetate	4.84E+08	65.22
	Ethyl acetate	1.28E+06	40.1

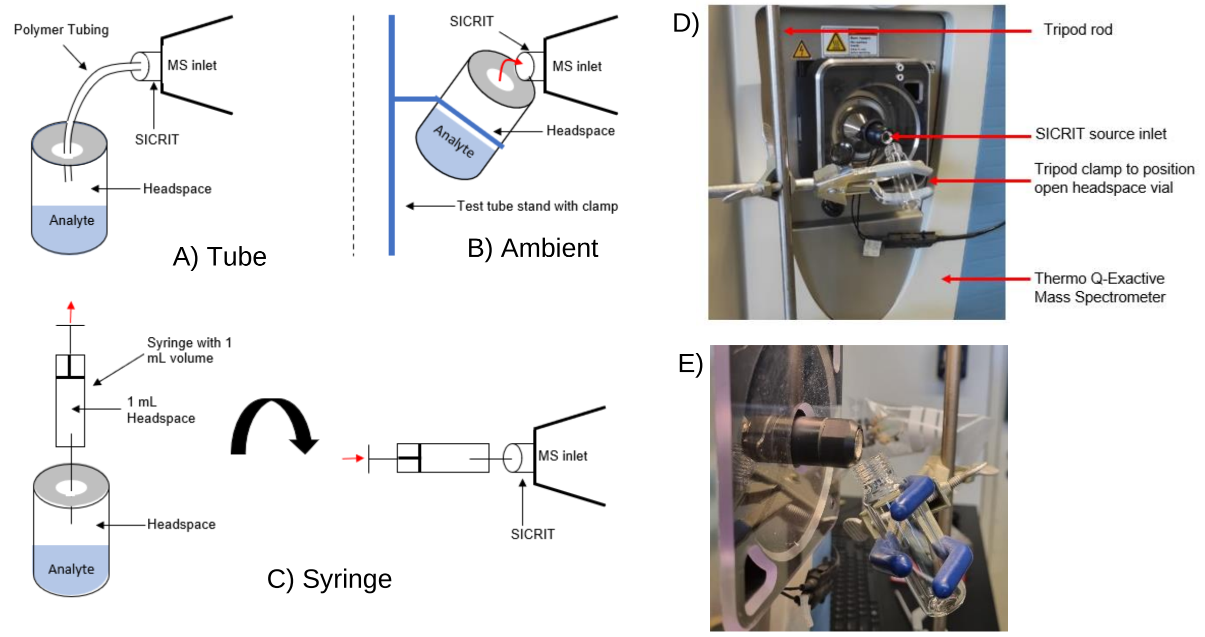

Figure S1. Different experimental setups tested during this study. A) Tube, b) Ambient, C) Syringe. D) Photo of ambient setup during analysis.

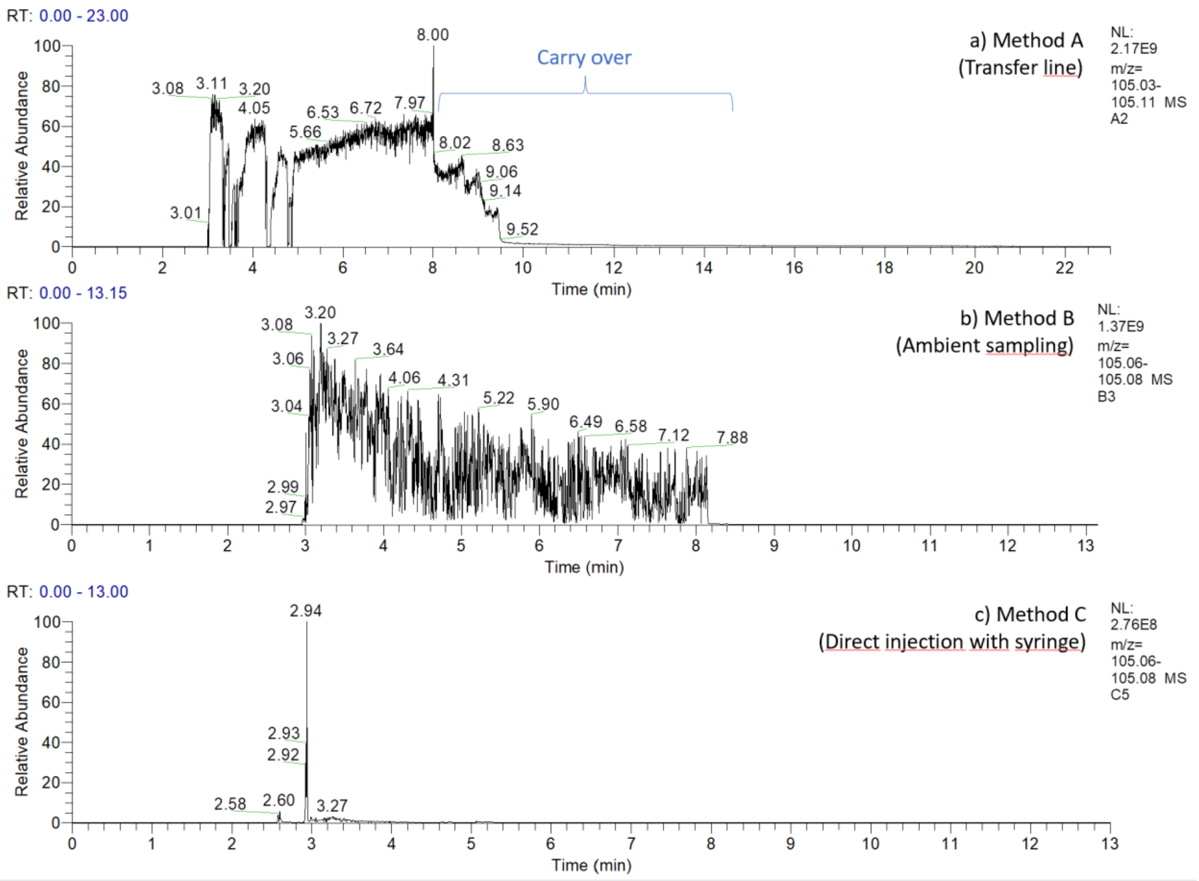

Figure S2. SIC for *m/z* 105, a common fragment of 2-phenyl ethanol and 2-phenethyl acetate for 3 different experimental setups, a) transfer line, b) ambient sampling, c) direct injection of the headspace with a syringe.

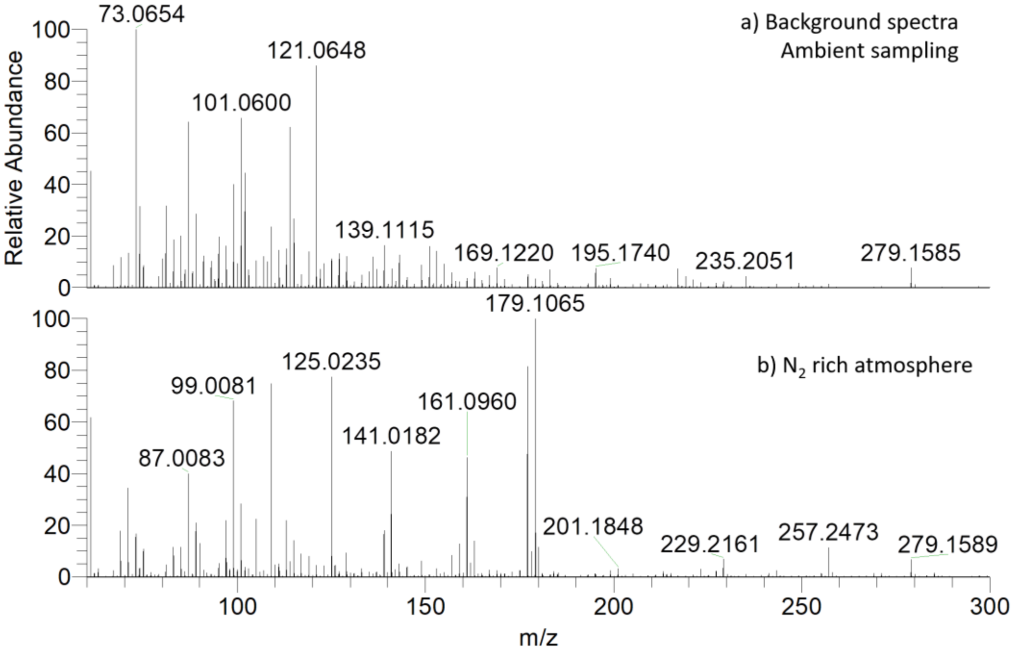

Figure S3. Background spectra for ambient and nitrogen-rich atmosphere.

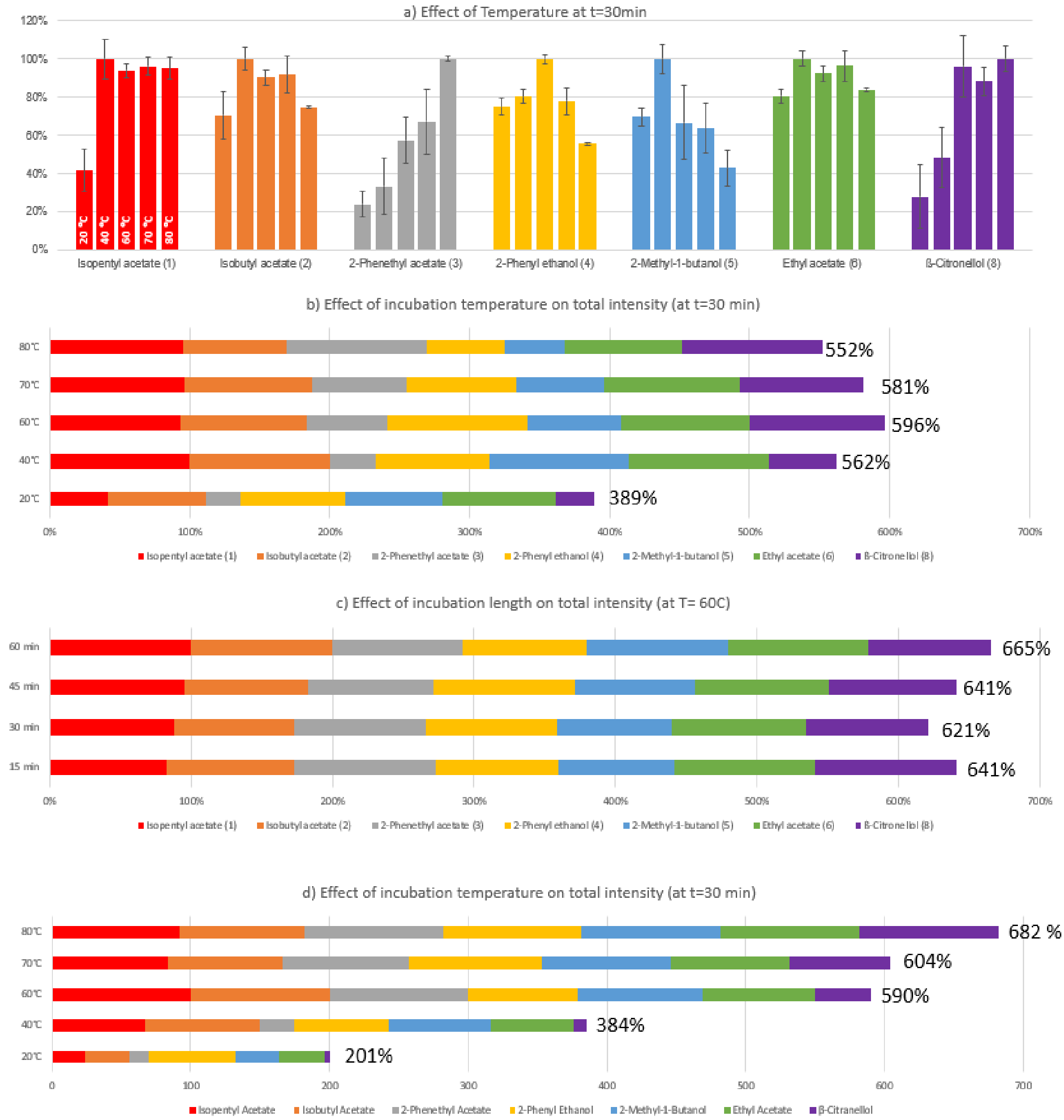

Figure S4. a) Effect of incubation temperature at t=30min for compounds 1-8. Effect of incubation temperature (b) and length (c) on the overall ion intensity for compounds **1**-**8**. d) Effect of incubation temperature at t=30min for compounds **1**-**8**. Citral (**7**) was excluded as it does not contain any unique *m/z* signals in comparison to β-citronellol (**8**). *m/z* values in Table 2 used for data extraction.

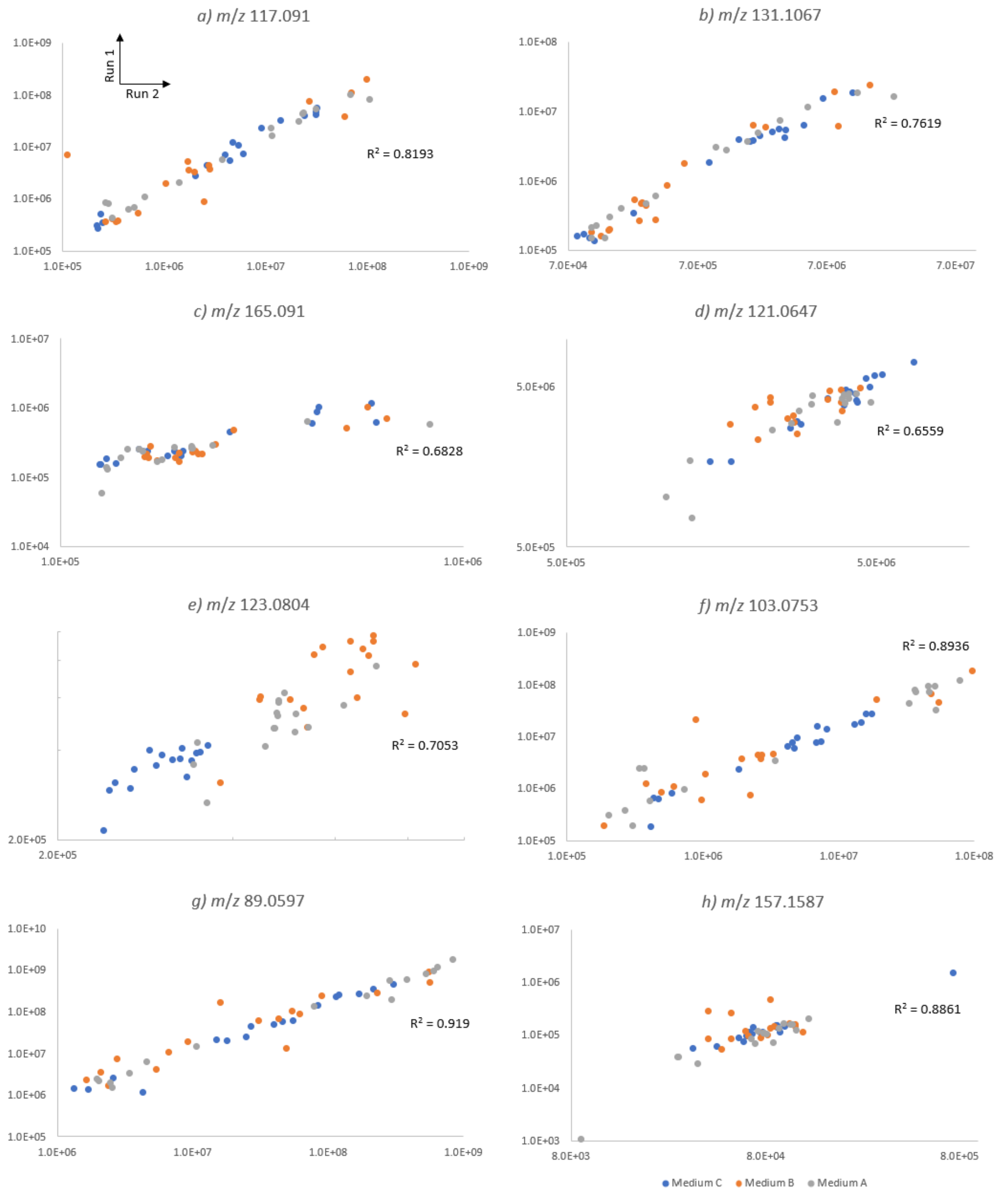

Figure S5. Correlation plots for two independent measurements (different day, different aliquot of same sample) on log-log scale for better visualisation of broad intensity ranges for a) *m/z* 117.091 (R^2 = 0.8193, derived from linear regression of linearly plotted data), b) *m/z* 131.1067 (R^2 = 0.7619), c) *m/z* 165.091 (R^2 = 0.6828), d) *m/z* 121.0647 (R^2 = 0.6559), e) *m/z* 123.0804 (R^2 = 0.7053), f) *m/z* 103.0753 (R^2 = 0.8936), g) *m/z* 89.0597 (R^2 = 0.919), h) *m/z* 157.1587 (R^2 = 0.8861). A larger spread of data can be seen for the Medium B (orange points) dataset compared to Medium A (grey) and C (blue).

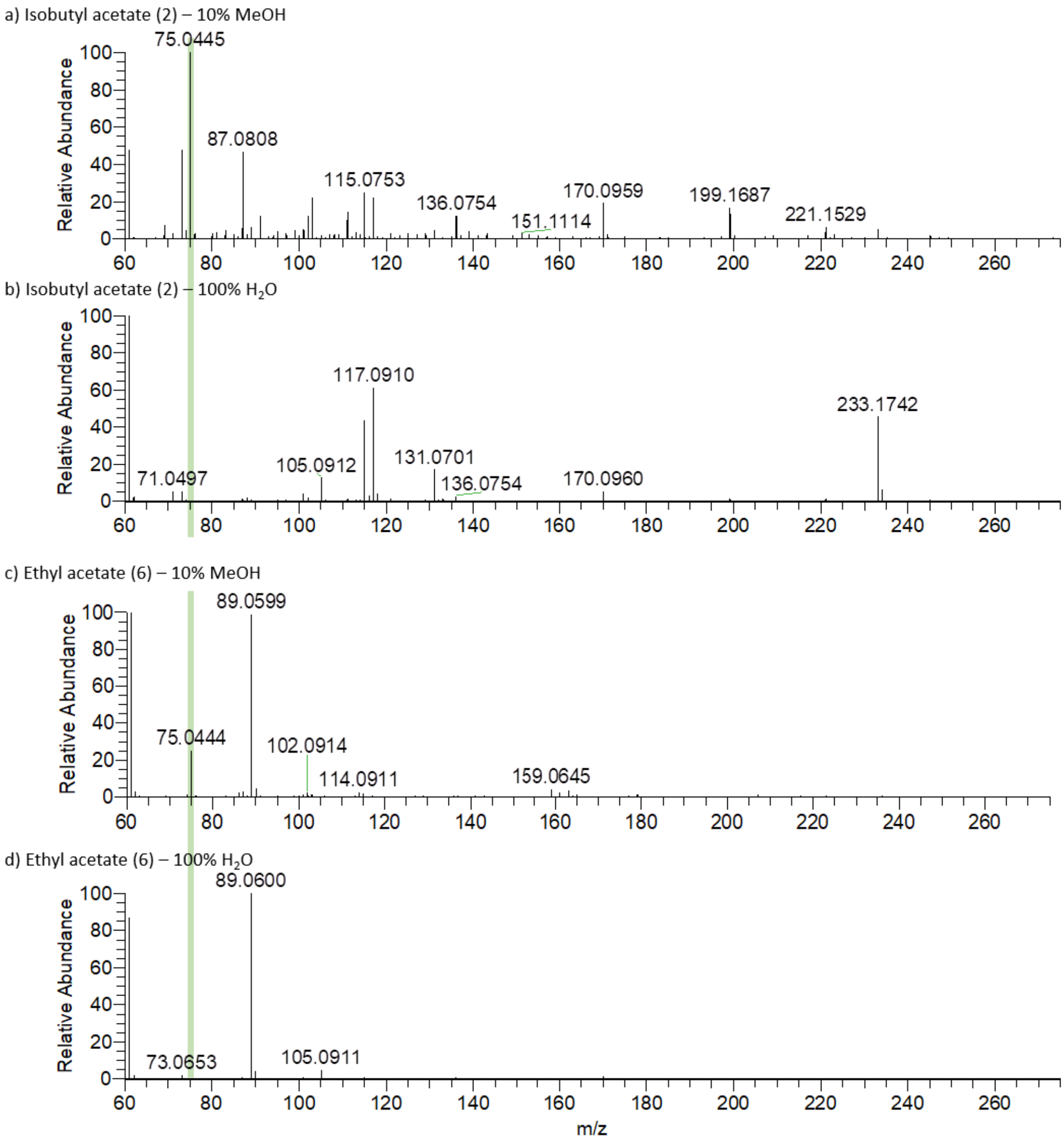

Figure S6. Transesterification with MeOH is observed if MeOH is added to the analyte solution prior to SICRIT analysis.

REFERENCES

(1) Lanza, E.; Ko, K. H.; Palmer, J. K. Aroma Production by Cultures of Ceratocystis Moniliformis. *J. Agric. Food Chem.* **1976**, *24* (6), 1247–1250. https://doi.org/10.1021/jf60208a019.

(2) Bluemke, W.; Schrader, J. Integrated Bioprocess for Enhanced Production of Natural Flavors and Fragrances by Ceratocystis Moniliformis. *Biomol. Eng.* **2001**, *17* (4–5), 137–142. https://doi.org/10.1016/S1389-0344(01)00072-7.

(3) Rottava, I.; Toniazzo, G.; Cortina, P. F.; Martello, E.; Grando, C. E.; Lerin, L. A.; Treichel, H.; Mossi, A. J.; de Oliveira, D.; Cansian, R. L.; Antunes, O. A. C.; Oestreicher, E. G. Screening of Microorganisms for Bioconversion of (−)β-Pinene and R-(+)-Limonene to α-Terpineol. *LWT - Food Sci. Technol.* **2010**, *43* (7), 1128–1131. https://doi.org/10.1016/j.lwt.2010.03.001.

(4) Rottava, I.; Cortina, P. F.; Martello, E.; Cansian, R. L.; Toniazzo, G.; Antunes, O. A. C.; Oestreicher, E. G.; Treichel, H.; de Oliveira, D. Optimization of α-Terpineol Production by the Biotransformation of R-(+)-Limonene and (−)-β-Pinene. *Appl. Biochem. Biotechnol.* **2011**, *164* (4), 514–523. https://doi.org/10.1007/s12010-010-9153-3.

(5) Li, M.; Xiao, Y.; Zhong, K.; Wu, Y.; Gao, H. Delving into the Biotransformation Characteristics and Mechanism of Steamed Green Tea Fermented by Aspergillus Niger PW-2 Based on Metabolomic and Proteomic Approaches. *Foods* **2022**, *11* (6), 865. https://doi.org/10.3390/foods11060865.

(6) de Carvalho, D. S.; Dionísio, A. P.; dos Santos, R.; Jr, S. B.; Godoy, H. T.; Pastore, G. M. Production of 1-Octen-3-Ol by Neurospora Species Isolated from Beiju in Different Culture Medium. *Procedia Food Sci.* **2011**, *1*, 1694–1699. https://doi.org/10.1016/j.profoo.2011.09.250.

(7) Pastore, G. M.; Park, Y. K.; Min, D. B. Production of Fruity Aroma by Neurospora from Beiju. *Mycol. Res.* **1994**, *98* (11), 1300–1302. https://doi.org/10.1016/S0953-7562(09)80302-X.
